# Supplementary material for: An Eleven-microRNA Signature Related to Tumor-Associated Macrophages Predicts Prognosis of Breast Cancer
Source: Int J Mol Sci. 2022 Jun 23;23(13):6994. doi: 10.3390/ijms23136994 (PMC9266835; doi:10.3390/ijms23136994)
Supplement: Supplementary file 1 [file ijms-23-06994-s001.zip › Supplementary Tables.pdf]

Supplementary Table S1: Pertinent clinicopathological parameters of the training, validation, and whole cohort of TCGA-BRCA

	Training set (n=856)	Validation set (n=207)	Whole cohort (n=1063)
**Age**			
Mean (SD)	58.6 (13.2)	57.3 (13.1)	58.3 (13.2)
Median [Min, Max]	58.0 [26.0, 90.0]	58.0 [29.0, 90.0]	58.0 [26.0, 90.0]
**Gender**			
Female	848 (99.1%)	203 (98.1%)	1051 (98.9%)
Male	8 (0.9%)	4 (1.9%)	12 (1.1%)
**Histological type**			
Invasive ductal carcinoma	613 (71.6%)	146 (70.5%)	759 (71.4%)
Invasive lobular carcinoma	159 (18.6%)	41 (19.8%)	200 (18.8%)
Mixed ductal and lobular carcinoma	21 (2.5%)	7 (3.4%)	28 (2.6%)
Others	62 (7.2%)	13 (6.3%)	75 (7.1%)
Missing	1 (0.1%)	0 (0%)	1 (0.1%)
**Pathological stage**			
I	141 (16.5%)	40 (19.3%)	181 (17.0%)
II	492 (57.5%)	109 (52.7%)	601 (56.5%)
III	198 (23.1%)	44 (21.3%)	242 (22.8%)
IV	13 (1.5%)	7 (3.4%)	20 (1.9%)
Missing	12 (1.4%)	7 (3.4%)	19 (1.8%)
**ER and HER2 status**			
ER+HER2-	450 (52.6%)	128 (61.8%)	578 (54.4%)
ER+HER2+	112 (13.1%)	19 (9.2%)	131 (12.3%)
ER-HER2+	30 (3.5%)	11 (5.3%)	41 (3.9%)
ER-HER2-	148 (17.3%)	23 (11.1%)	171 (16.1%)
Missing	116 (13.6%)	26 (12.6%)	142 (13.4%)
**PAM50**			
Normal	17 (2.0%)	5 (2.4%)	22 (2.1%)
Luminal A	331 (38.7%)	82 (39.6%)	413 (38.9%)
Luminal B	137 (16.0%)	45 (21.7%)	182 (17.1%)
HER2	51 (6.0%)	12 (5.8%)	63 (5.9%)
Basal	112 (13.1%)	20 (9.7%)	132 (12.4%)
Missing	208 (24.3%)	43 (20.8%)	251 (23.6%)

Supplementary Table S2: Relationship between risk score and pertinent clinicopathological parameters in TCGA-BRCA cohort

		Risk score		
	**n**	**Mean (SD)**	***p***	**test**
**Age**	1063		<0.001	Pearson correlation *r*=0.1064
**Gender**				
Female	1051	8.48 (0.524)	<0.001	Welch t-test
Male	12	9.02 (0.300)		
**Histological type**				
Invasive ductal carcinoma	759	8.51 (0.523)	<0.001	ANOVA test
Invasive lobular carcinoma	200	8.36 (0.466)		
Mixed ductal and lobular carcinoma	28	8.39 (0.461)		
Others	75	8.61 (0.647)		
**Pathological stage**				
I	181	8.39 (0.468)	0.0135	ANOVA test
II	601	8.48 (0.551)		
III	242	8.55 (0.487)		
IV	20	8.62 (0.472)		
**ER and HER2 Status**				
ER+HER2-	578	8.43 (0.484)	0.006	ANOVA test
ER+HER2+	131	8.55 (0.476)		
ER-HER2+	41	8.65 (0.668)		
ER-HER2-	171	8.51 (0.641)		
**PAM50**				
Normal	22	8.04 (0.441)	<0.001	ANOVA test
Luminal A	413	8.33 (0.461)		
Luminal B	182	8.63 (0.470)		
HER2	63	8.59 (0.535)		
Basal	132	8.43 (0.610)		
