# Supplementary figures and images for: An Eleven-microRNA Signature Related to Tumor-Associated Macrophages Predicts Prognosis of Breast Cancer
Source: Int J Mol Sci. 2022 Jun 23;23(13):6994. doi: 10.3390/ijms23136994 (PMC9266835; doi:10.3390/ijms23136994)

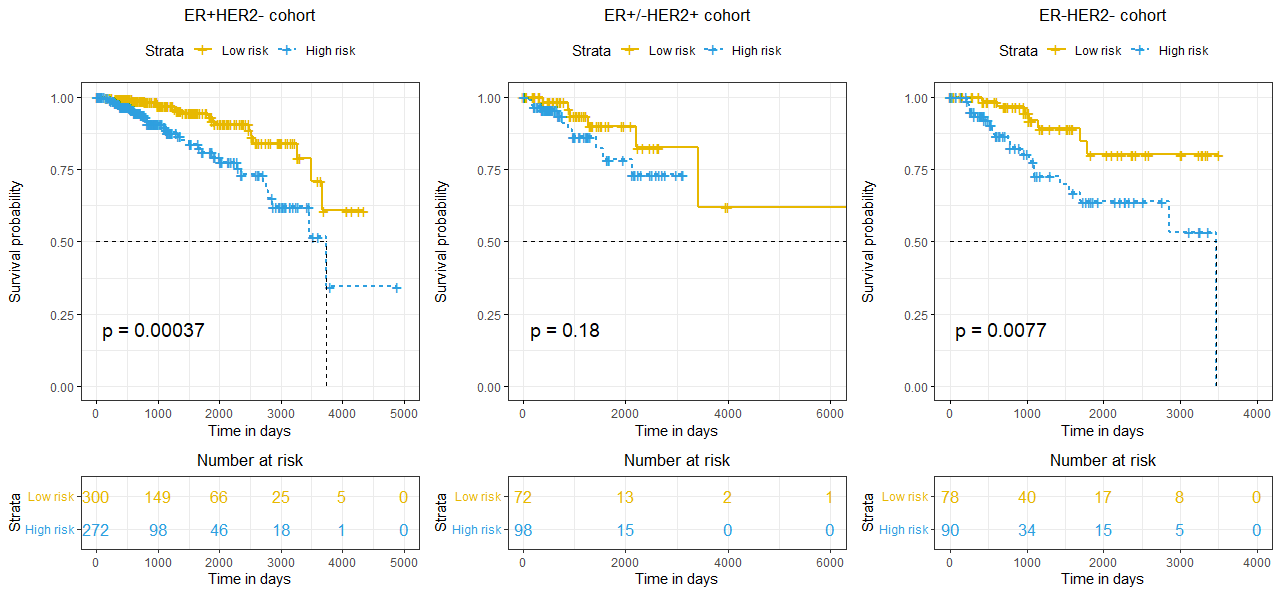

Supplement: Supplementary file 1 [file ijms-23-06994-s001.zip › Suplementary figure S2.tiff]

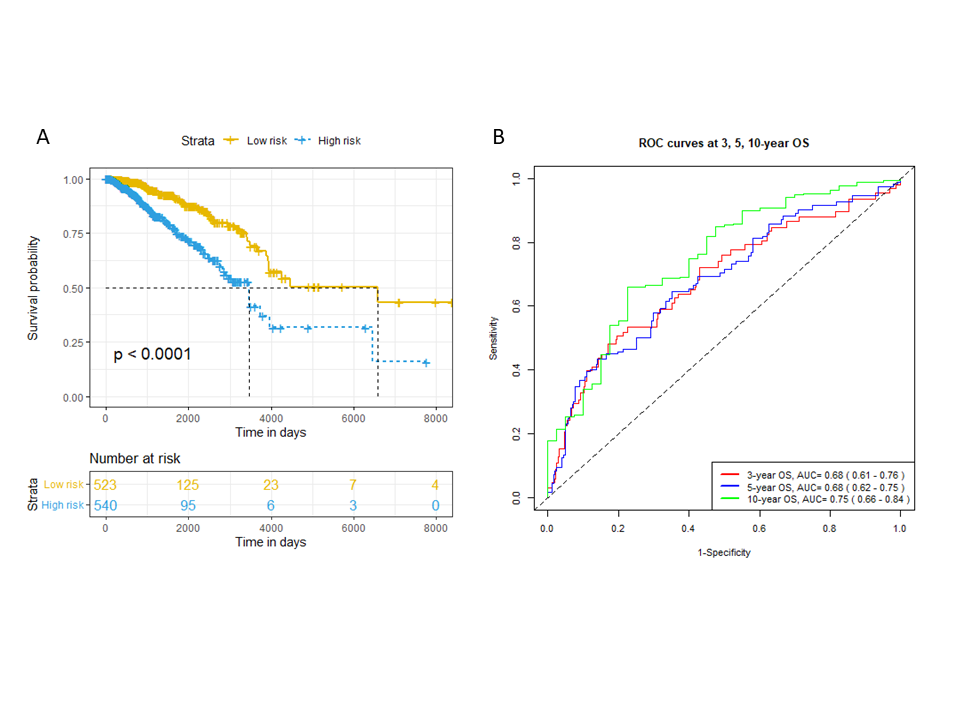

Supplement: Supplementary file 1 [file ijms-23-06994-s001.zip › Supplementary figure S1.tif]

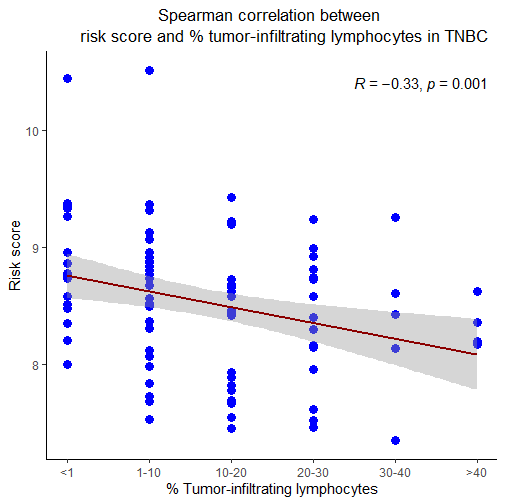

Supplement: Supplementary file 1 [file ijms-23-06994-s001.zip › Supplementary figure S3.tiff]

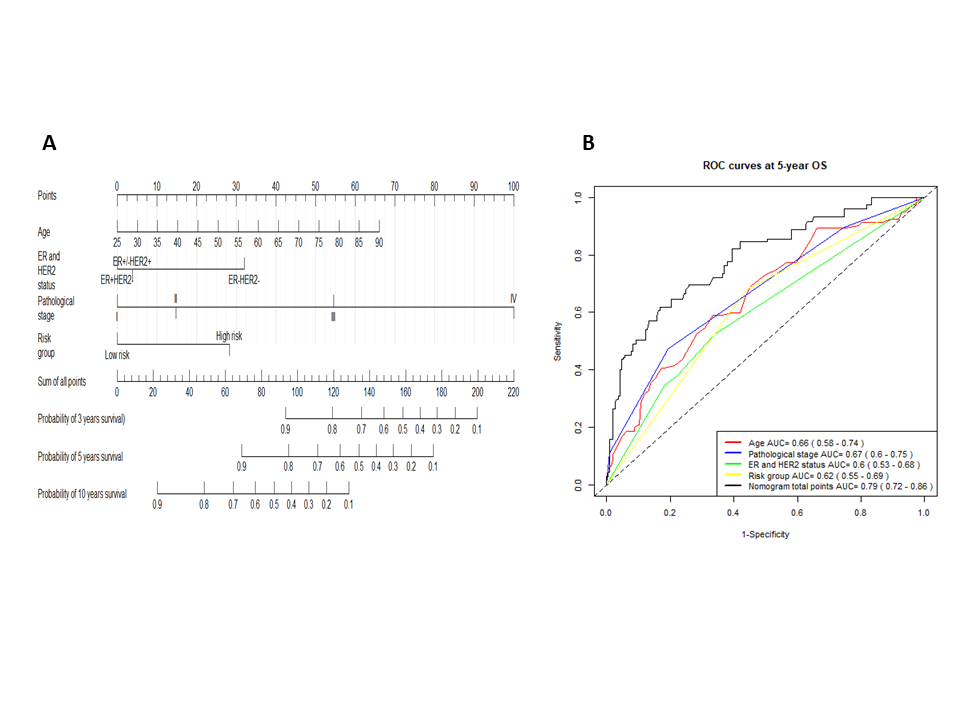

Supplement: Supplementary file 1 [file ijms-23-06994-s001.zip › Supplementary figure S4.tif]

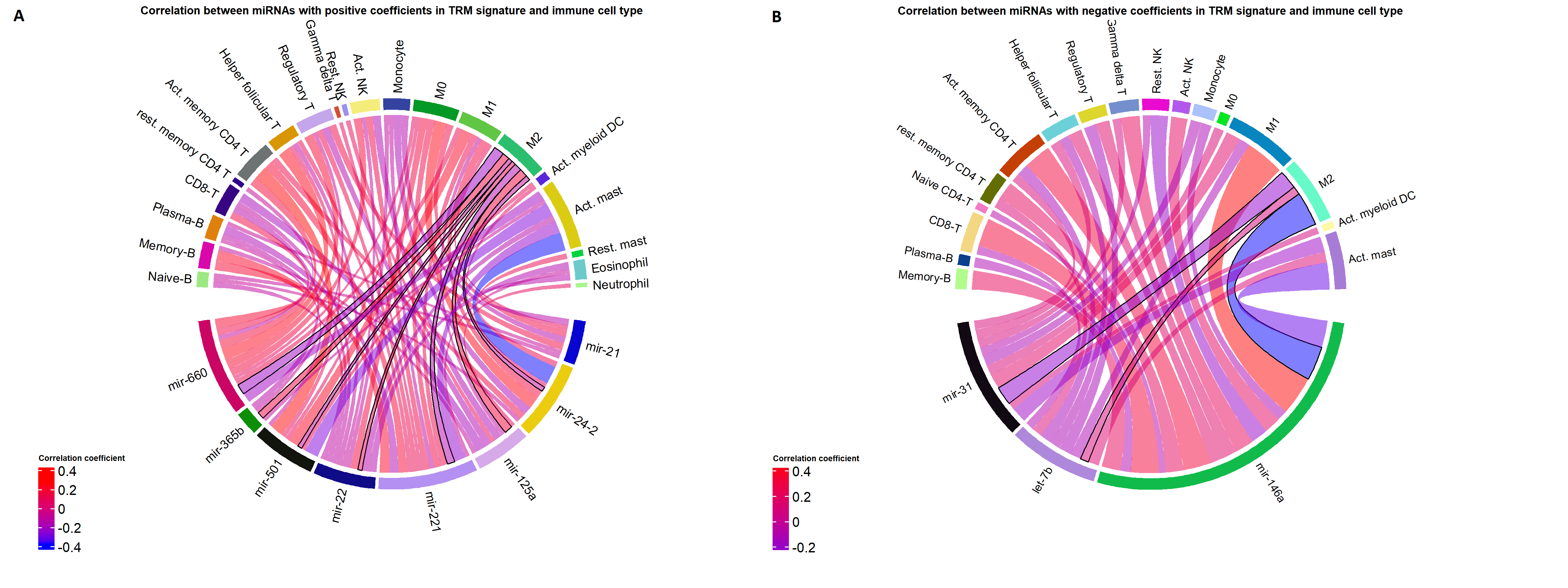

Supplement: Supplementary file 1 [file ijms-23-06994-s001.zip › Supplementary figure S5.tif]

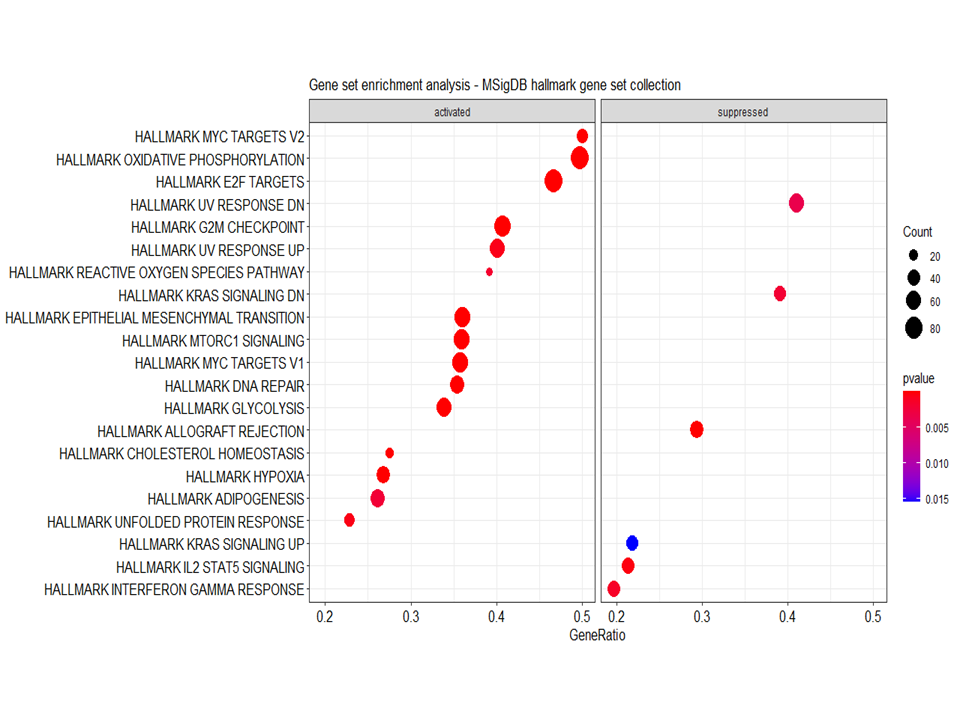

Supplement: Supplementary file 1 [file ijms-23-06994-s001.zip › Supplementary figure S6.tif]
